# Differences in diagnosis, management, and outcomes of acute febrile illness by health facility level in southern Ethiopia

**DOI:** 10.1038/s41598-022-23641-8

**Published:** 2022-11-10

**Authors:** Techalew Shimelis, Susana Vaz Nery, Gill Schierhout, Birkneh Tilahun Tadesse, Sabine Dittrich, John A. Crump, John M. Kaldor

**Affiliations:** 1 Kirby Institute, University of New South Wales, Sydney, Australia; 2 College of Medicine and Health Sciences, Hawassa University, Hawassa, Ethiopia; 3 The George Institute for Global Health, University of New South Wales, Sydney, Australia; 4 Foundation for Innovative New Diagnostics, Geneva, Switzerland; 5 Nuffield Department of Medicine, University of Oxford, Oxford, UK; 6 Centre for International Health, University of Otago, Dunedin, New Zealand

**Keywords:** Microbiology, Diseases, Medical research, Signs and symptoms

## Abstract

We assessed the diagnosis, management and outcomes of acute febrile illness in a cohort of febrile children aged under 5 years presenting at one urban and two rural health centres and one tertiary hospital between 11 August 2019 and 01 November 2019. Pneumonia was diagnosed in 104 (30.8%) of 338 children at health centres and 128 (65.0%) of 197 at the hospital (p < 0.001). Malaria was detected in 33 (24.3%) of 136 children at the urban health centre, and in 55 (55.6%) of 99 and 7 (7.4%) of 95 children at the rural health centres compared to 11 (11.6%) of 95 at the hospital. Antibacterials were prescribed to 20 (11.5%) of 174 children without guidelines-specified indications (overprescribing) at health centres and in 7 (33.3%) of 21 children at the hospital (p = 0.013). Antimalarials were overprescribed to 13 (7.0%) of 185 children with negative malaria microscopy at the hospital. The fever resolved by day 7 in 326 (99.7%) of 327 children at health centres compared to 177 (93.2%) of 190 at the hospital (p < 0.001). These results suggest that additional guidance to health workers is needed to optimise the use of antimicrobials across all levels of health facilities.

## Introduction

Continuing the substantial reduction of childhood mortality in low-resource settings requires a focus on improved diagnosis and management of infectious diseases, many of which present commonly with fever, across all levels of the health service^1–3^. To assist health workers in responding to childhood febrile illnesses in these settings, simplified World Health Organization (WHO) guidelines are available, including the Pocket Book of Hospital Care for use at hospitals^4^ and the integrated management of childhood illness (IMCI) at health centres^5^. These guidelines recommend prescribing antibacterial therapy based on meeting clinical case definitions for illnesses, including pneumonia and dysentery, and emphasize parasitological confirmation of malaria before offering antimalarial agents. Integrating malaria diagnostics, including microscopy and rapid diagnostic tests, into fever case management has reduced the unnecessary use of antimalarials^6^, and contributed to the substantial global reduction in malaria morbidity and mortality^7,8^. However, a lack of accessible diagnostics for various bacterial and viral infections has remained a barrier to improved guidelines and practice in fever management^9^. For fever without clinical evidence of bacterial infection, guidelines recommend withholding antibacterial treatment unless patients have signs of severe illness^10^. Nevertheless, in the absence of reliable diagnostics to explain the cause of the fever in patients with negative malaria tests, health workers may not adhere to guidelines and unnecessarily prescribe antimalarials^11–13^ and antibacterials^14,15^, which may lead to the development of drug resistance^16,17^.

We recently reported a low proportion of malaria (3.2%)^18^, a lack of adherence to guidelines in prescribing antimicrobials (antibacterials and antimalarials), and substantial proportions of hospitalization (38.1%) and in-hospital fatality ratio (5.9%) in febrile children aged under 13 years presenting at the largest tertiary hospital in southern Ethiopia^19^. To determine how the pattern of childhood febrile illness, health workers’ adherence to guidelines for the initiation of antimicrobials, and outcomes may differ in other health settings, we extended our investigation to lower-level health facilities in the catchment areas of the hospital. Thus, the current study aimed to assess whether there are differences in the diagnosis, management, and outcomes of febrile illness in children under 5 years old presenting at lower-level of health facilities (urban and rural health centres) compared to the tertiary hospital.

## Methods

### Study design and setting

**Figure 1:**
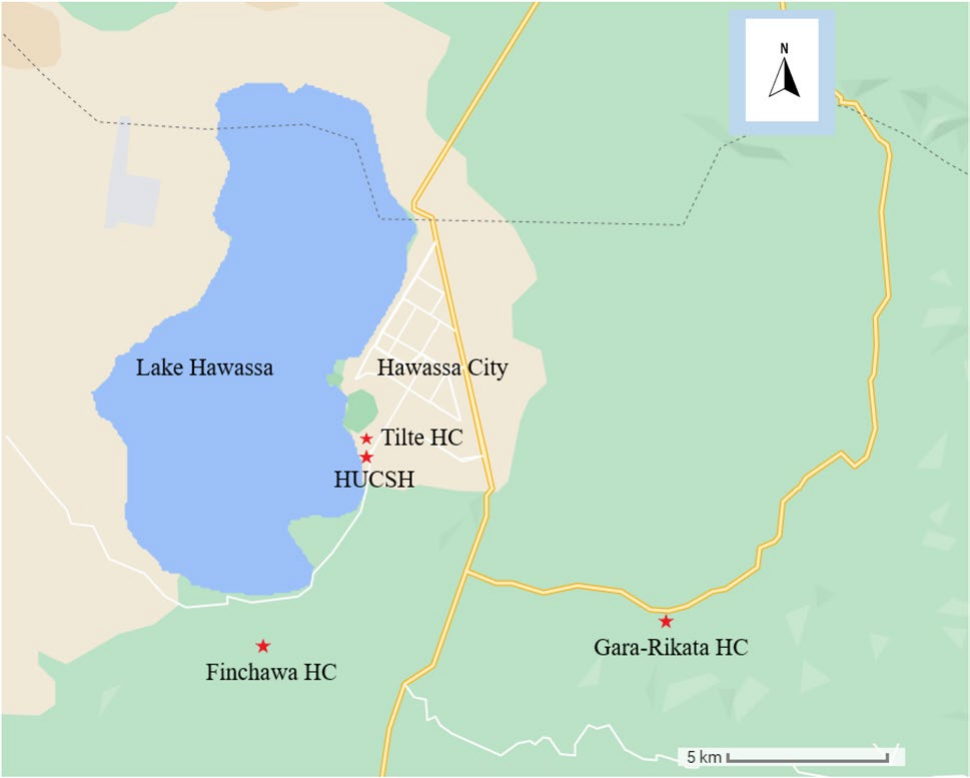
Study sites, Hawassa City, 2019. *HC* health centre, *HUCSH* Hawassa University Comprehensive Specialized Hospital.

This study is reported according to the Strengthening the Reporting of Observational Studies in Epidemiology (STROBE) guideline (Checklist S1). A prospective cohort study was undertaken from 11 August 2019 to 01 November 2019 at health facilities in and around Hawassa City, southern Ethiopia, located on the shores of Lake Hawassa, the capital of Southern Nations, Nationalities, and Peoples’ Region (SNNPR). Tilte health centre from an urban area and Finchawa and Gara-Rikata health centres from rural areas were lower-level health facilities purposively selected for the study, considering the distance from Lake Hawassa, which may influence water-associated infectious diseases. We also recruited participants from Hawassa University Comprehensive Specialized Hospital (HUCSH), the largest tertiary hospital in the SNNPR. As shown in Fig. 1, all health facilities are close to Lake Hawassa, except Gara-Rikata, which is located about 10 km away. The major malaria transmission season in Ethiopia is from September to December, following the rainy season during June to August. About 65% of the administrative region of SNNPR is malarious though the incidence has declined in recent years^20^. In addition to vaccines for tuberculosis, diphtheria, pertussis, tetanus, poliomyelitis, measles, and hepatitis B virus, the national childhood immunization program in Ethiopia introduced the *Haemophilus influenzae* type b vaccine, pneumococcal conjugate vaccine, and monovalent rotavirus vaccine more recently^21^. Full immunization (as defined by the Ethiopian vaccination schedule) coverage of SNNPR among children aged 12–23 months was 38% in 2019^22^.

### Participant eligibility and enrolment

Children consecutively presenting to the participating health facilities to seek care 7 days per week, 24 h per day, during the study period were screened for study eligibility. Those children under 5 years old with fever, defined as an axillary temperature of at least 37.5 °C or a history of fever at least once in the past 48 h, lasting no longer than 7 days, were enrolled. We excluded patients for whom minor skin infections were the main reasons for the visit, and those who were referred to a hospital urgently, therefore, did not undergo routine examinations and management at health centres.

### Sample size

The sample size was computed based on outcomes reported in another study, relating to health-seeking behaviour by caregivers attending various levels of health facilities^23^. Thus, the current analysis was based on data gathered from the 338 enrolled children from health centres, 138 from Tilte and 100 each from Finchawa and Gara-Rikata, and 197 from the hospital. The target sample size for each facility was proportional to the number of patients recorded as having attended in the same season in the previous year. Consecutive febrile children meeting the inclusion criteria were enrolled until the targeted sample size was obtained.

### Data collection

#### Diagnosis and management on enrolment day

The study personnel were nurses and health officers from each facility, who had 4 years of training in clinical services. They received training in the study data collection procedures, and reviewed patients’ records immediately on initial clinical management on enrolment day. Data captured using a study-specific form included the child’s demographic characteristics (sex, age), clinical information (treatment taken prior to study enrolment for index illness, duration of fever, axillary temperature at presentation, and symptoms and signs) and laboratory and clinical diagnosis. The management done for the participants at the study facilities, including treatment prescribed, hospital admission, and referral to higher-level care, were also captured. Available at all participating health facilities were blood smear microscopy for haemoparasites and stool microscopy for intestinal parasites. Thick and thin blood smear slides were stained with Giemsa and examined for blood parasites (*Plasmodium* species and other haemoparasites) by experienced Laboratory Technologists with a 4-year training degree. Stool samples were collected from patients with gastrointestinal symptoms and processed using direct microscopy (saline and iodine mounts) to detect intestinal parasites. We adopted case definitions for presenting conditions diagnosed following the Ethiopian Ministry of Health guidelines (case definitions in Table 1)^24,25^. Malaria was defined as positive blood smear microscopy for *Plasmodium* species. Fever without an identified source of infection based on clinical and laboratory investigations conducted was defined as undifferentiated fever (Table 1). Based on clinicians’ decisions, patients presenting to the hospital were managed as outpatients or inpatients. Likewise, health centre staff managed children as outpatients or referred them to higher-level care.

**Table 1:** Study definitions^19^.

**Illnesses**	
Acute respiratory infection	Presentation with at least one respiratory sign or symptom of less than 14 days and localized to the respiratory tract (upper or lower)
Pneumonia	A history of cough and/or difficult breathing, plus signs of (a) fast breathing OR (b) chest findings
Acute tonsillopharyngitis	Presentation with (a) pharyngeal redness and enlarged tonsils or (b) neck lymph node and enlarged tonsils or (c) tonsillar exudate, which are suggestive of bacterial infection based on the national guidelines
Unspecified upper respiratory tract infections	Presentation with at least one respiratory sign or symptom (e.g. cough, rhinorrhoea) in the absence of features consistent with other specified respiratory illnesses
Acute diarrhoea	Presentation with diarrhoea (stool frequency > 3 loose or liquid stools per day on at least one day in the week prior to enrolment) lasting less than 14 days^24^
Sepsis	The presence of systemic inflammation response syndrome with suspected or proven infection, or with some form of organ dysfunction
Meningitis	Presentation with a stiff neck or positive meningeal signs diagnosed by clinicians as a case of meningitis on enrolment day
Malaria	A positive blood smear microscopy for the asexual stages of Plasmodium species
Undifferentiated fever	Cases without identified source of infection for the fever on clinical and laboratory investigations conducted
**Outcomes**	
Resolved fever	Absence of fever for 2 consecutive days prior to day 7 (± 1) as reported by caregivers or measured temperature of 36.4 °C − 37.5 °C
Persisting fever	Fever episode within 2 days prior to day 7 (± 1) as reported by caregivers or measured temperature of ≥ 37.5 °C
Hospitalization	Admission to hospital for treatment in relation to the presenting febrile illness
Death	Mortality within 7 (± 1) days follow-up period, and potentially linked to the presenting febrile illness as judged by attending clinicians

#### Health workers’ prescribing adherence with management guidelines

Two senior paediatricians at HUCSH defined illnesses requiring antibacterial therapy based on guidelines^24,25^, as summarised in Table S1. The prescribing of antibacterials without clinical indication based on guidelines, and prescribing antimalarials without confirmation of malaria (tested negative or without having a blood test) were defined as overprescribed.

#### Follow-up data

Clinical outcome data were collected from caregivers of children on day 7 (± 1) using a study- specific form. Caregivers of children managed as outpatients were contacted by telephone or visited at home in rural areas if contact by telephone was impossible. For hospitalized patients, records were also reviewed to collect follow-up data. Outcomes assessed included resolution of fever, hospital stay, hospitalization after initial management, re-consultation at the same facility or visit to other facilities, and death.

### Data analysis

Data were entered using EpiData version-3.1^26^, and analysed using SPSS Statistics for Windows, Version 20.0 (IBM SPSS Statistics for Windows, Version 20.0; IBM Corp., Armonk, NY, United States). Results for categorical variables, including demographic and clinical characteristics, diagnosed illnesses, treatment, and outcomes, were summarized using frequency (percentage). Quantitative data, including child age, duration of fever before and after enrolment, and hospital stay, were summarized using median (interquartile range, IQR). Differences between proportions of demographic and clinical characteristics, diagnosed illnesses, prescribed treatments and adherence with guidelines, and outcomes at lower-level health facilities (health centres) and a higher-level facility (tertiary hospital) were evaluated using the Pearson Chi-square test or Fisher’s Exact test as appropriate. A p-value < 0.05 from any statistical test was considered to show a significant difference.

### Ethics approval and consent to participate

The study was approved by the Human Research Ethics Committee of the University of New South Wales (UNSW), Sydney, Australia (Ref. No: HC190358) and the Institutional Review Board of Hawassa University College of Medicine and Health Sciences, Hawassa, Ethiopia (Ref. No: IRB/223/11). Adequate information was given to caregivers about the study. Participation was voluntary, and informed written consent was obtained from caregivers. All methods were carried out following relevant guidelines and regulations.

## Results

### Demographic characteristics of the children

Overall, 535 participants were enrolled in the study, 138 (25.8%) from the urban health centre (Tilte), 100 (18.7%) each from the rural health centres (Finchawa and Gara-Rikata), and 197 (36.8%) at the tertiary hospital, HUCSH. A further 9 children were determined to have not met the eligibility criteria, so they were excluded. On average, children recruited at HUCSH were younger than those at health centres, with median age (IQR) of 12 (6–24) months and 24 (12–36) months, respectively, p < 0.001 (Table 2). No significant difference was observed in the distribution of child’s sex between health centres and the hospital (p = 0.608) (Table 2).

**Table 2:** Demographic and clinical characteristics of febrile children by study health facilities in Hawassa City, 2019.

Characteristics	Health centres				HUCSH n (%) (N = 197)	p-value^¶^
	Tilte HC n (%) N = 138	Finchawa HC n (%) N = 100	Gara-Rikata HC n (%) N = 100	Total (HCs) n (%) (N = 338)		
**Age (month)**						
≤ 11	33 (23.9)	21 (21.0)	26 (26.0)	80 (23.7)	96 (48.7)	< 0.001*
12–23	32 (23.2)	20 (20.0)	20 (20.0)	72 (21.3)	51 (25.9)	
24–35	26 (18.8)	19 (19.0)	22 (22.0)	67 (19.8)	26 (13.2)	
36–47	31 (22.5)	25 (25.0)	21 (21.0)	77 (22.8)	12 (6.1)	
48–59	16 (11.6)	15 (15.0)	11 (11.0)	42 (12.4)	12 (6.1)	
**Sex**						
Male	67 (48.6)	65 (65.0)	61 (61.0)	193 (57.1)	108 (54.8)	0.608
Female	71 (51.4)	35 (35.0)	39 (39.0)	145 (42.9)	89 (45.2)	
**Clinical history**						
Treatment prior to enrolment for present episode						
Antibacterials	4 (2.9)	2 (2.0)	5 (5.0)	11 (3.3)	103 (52.3)	< 0.001*
Antimalarials	0	0	2 (2.0)	2 (0.6)	8 (4.1)	0.006**
Duration of fever						
1 day	104 (75.4)	41 (41.0)	40 (40.0)	185 (54.7)	34 (17.3)	< 0.001*
2 - 4 days	30 (21.7)	54 (54.0)	59 (59.0)	143 (42.3)	104 (52.8)	
5 - 7 days	4 (2.9)	5 (5.0)	1 (1.0)	10 (3.0)	59 (29.9)	
Axillary temperature						
< 37.5 °C	36 (26.1)	10 (10.0)	35 (35.0)	81 (24.0)	32 (16.2)	< 0.001*
37.5 - 38.9 °C	77 (55.8)	61 (61.0)	50 (50.0)	188 (55.6)	149 (75.6)	
≥ 39.0 °C	25 (18.1)	29 (29.0)	15 (15.0)	69 (20.4)	16 (8.1)	
**Most frequently reported signs/symptoms**						
Cough	55 (39.9)	48 (48.0)	42 (42.0)	145 (42.9)	151 (76.6)	< 0.001*
Fast breathing	31 (22.5)	32 (32.0)	33 (33.0)	96 (28.4)	113 (57.4)	< 0.001*
Vomiting	39 (28.3)	37 (37.0)	14 (14.0)	90 (26.6)	88 (44.7)	< 0.001*
Shivering	26 (18.8)	37 (37.0)	4 (4.0)	67 (19.8)	3 (1.5)	< 0.001*
Tonsillar signs	45 (32.6)	2 (2.0)	5 (5.0)	52 (15.4)	4 (2.0)	< 0.001*
Diarrhoea	16 (11.6)	14 (14.0)	11 (11.0)	41 (12.1)	36 (18.3)	0.051
Excessive sweating	0	23 (23.0)	1 (1.0)	24 (7.1)	21 (10.7)	0.153
Loss of appetite	14 (10.1)	3 (3.0)	2 (2.0)	19 (5.6)	19 (9.6)	0.081
Abdominal pain	3 (2.2)	0	12 (12.0)	15 (4.4)	11 (5.6)	0.552
Runny nose	5 (3.6)	0	2 (2.0)	7 (2.1)	12 (6.1)	0.015*
Refuse to drink or breastfeed	0	3 (3.0)	1 (1.0)	4 (1.2)	19 (9.6)	< 0.001*
Grunting	1 (0.7)	2 (2.0)	0	3 (0.9)	27 (13.7)	< 0.001*
Multiple signs and symptoms						
One	8 (5.8)	6 (6.0)	16 (16.0)	30 (8.9)	2 (1.0)	< 0.001*
Two	44 (31.9)	29 (29.0)	41 (41.0)	114 (33.7)	21 (10.7)	
Three	56 (40.6)	35 (35.0)	39 (39.0)	130 (38.5)	67 (34.0)	
Four	24 (17.4)	25 (25.0)	3 (3.0)	52 (15.4)	64 (32.5)	
Five and above	6 (4.3)	5 (5.0)	1 (1.0)	12 (3.6)	43 (21.8)	

*HC* health centre, *HUCSH* Hawassa University Comprehensive Specialized Hospital.

^**¶**^Comparison of proportions between all health centres and HUCSH.

*Significant difference (p-value < 0.05 from chi-square test).

**Significant difference (p-value < 0.05 from Fisher’s exact test).

### Presenting clinical characteristics

The median (IQR) duration of fever at enrolment among children attending health centres was 1 (1–2) day compared to 3 (2–5) days in those presenting to HUCSH. Of 338 children attending health centres, 11 (3.3%) had taken antibacterial agents for the current episode of illness prior to enrolment compared to 103 (52.3%) of 197 at HUCSH (p < 0.001). Further, lower proportions of children attending health centres compared to HUCSH had a cough (42.9% vs. 76.6%; p < 0.001), fast breathing (28.4% vs. 57.4%), and vomiting (26.6% vs. 44.7%). In contrast, tonsillar signs and shivering were more common presenting features among children at health centres (p < 0.001) (Table 2). Children presenting at lower-level health facilities had a smaller number of symptoms and signs compared to those at a tertiary hospital (p < 0.001).

### Diagnoses

As shown in Table 3, 180 (53.3%) of 338 children at health centres compared to 150 (76.1%) of 197 children at HUCSH had acute respiratory infections (ARIs). Pneumonia was a common illness, diagnosed in 104 (30.8%) of children at health centres and in 128 (65.0%) of those at HUCSH (p < 0.001). Acute tonsillopharyngitis was more frequent at Tilte urban health centre (HC), diagnosed in 46 (33.3%) of 138 children. Malaria was found in 95 (28.8%) of 330 children whose blood samples were examined by microscopy at health centres, and in 11 (11.6%) of 95 children at HUCSH. At Finchawa rural HC, 55 (55.6%) of 99 children had malaria compared to 7 (7.4%) of 95 at Gara-Rikata rural HC, and 33 (24.3%) of 136 at Tilte urban HC. Of 425 blood samples tested at all study sites, 48 (11.3%) were positive for *Plasmodium falciparum* and 60 (14.1%) for *P. vivax*; with 2 (0.5%) mixed infections. Thirty-nine (11.6%) of 338 children at health centres and 8 (4.1%) of 197 children at HUCSH had undifferentiated fever (p = 0.003).

**Table 3:** Diagnosed infectious conditions in febrile children by study health facilities in Hawassa City, 2019.

Infectious conditions diagnosed	Health centres				HUCSH n (%) (N = 197)	p-value^¶^
	Tilte HC n (%) N = 138	Finchawa HC n (%) N = 100	Gara-Rikata HC n (%) N = 100	Total (HCs) n (%) (N = 338)		
**Acute respiratory infections**	92 (66.7)	39 (39.0)	49 (49.0)	180 (53.3)	150 (76.1)	< 0.001*
Acute tonsillopharyngitis	46 (33.3)	2 (2.0)	5 (5.0)	53 (15.7)	5 (2.5)	< 0.001*
Unspecified URTIs	8 (5.8)	5 (5.0)	10 (10.0)	23 (6.8)	13 (6.6)	0.927
Pneumonia	38 (27.5)	32 (32.0)	34 (34.0)	104 (30.8)	128 (65.0)	< 0.001*
Other LRTIs	0 (0)	0 (0)	0 (0)	0 (0)	4 (2.0)	–
Acute diarrhoea	16 (11.6)	14 (14.0)	11 (11.0)	41 (12.1)	36 (18.3)	0.051
Meningitis	0 (0)	0 (0)	0 (0)	0 (0)	20 (10.2)	–
Sepsis	0 (0)	0 (0)	0 (0)	0 (0)	11 (5.6)	–
**Malaria**^**ℼ**^	33 (24.3)^a^	55 (55.6)^b^	7 (7.4)^c^	95 (28.8)^d^	11 (11.6)^e^	0.001*
*P. falciparum* infection	22 (16.2)^a^	21 (21.2)^b^	3 (3.2)^c^	46 (13.9)^d^	2 (2.1)^e^	0.001*
*P. vivax* infection	11 (8.1)^a^	35 (35.4)^b^	5 (5.3)^c^	51 (15.5)^d^	9 (9.5)^e^	0.140
Intestinal parasitosis^**ℼ**^	2 (20.0)^f^	1 (-)	13 (76.5)^g^	16 (57.1)^k ‡^	4 (12.9)^m †^	< 0.001*
Other infectious conditions	3 (2.2)	0 (0)	2 (2.0)	5 (1.5)^§^	11 (5.6)^δ^	–
Undifferentiated fever^**^	11 (8.0)	5 (5.0)	23 (23.0)	39 (11.5)	8 (4.1)	0.003*

*HC* health centre, *HUCSH* Hawassa University Comprehensive Specialized Hospital, *URTIs* upper respiratory tract infections, *LRTIs* lower respiratory infections.

^**ℼ**^Diagnosis made based on laboratory findings.

Among children whose blood samples were examined by microscopy: ^a^(N = 136); ^b^(N = 99); ^c^(N = 95); ^d^(N = 330); ^e^(N = 95).

Among children whose stool samples were examined by microscopy: ^f^(N = 10); ^g^(N = 17); ^k^(N = 28); ^m^(N = 31).

^†^Giardiasis (n = 1), ascariasis (n = 2), isosporiasis (n = 1).

^‡^Giardiasis (n = 3), ascariasis (n = 12), trichuriasis (n = 1).

^δ^Acute otitis media (n = 1), eye infection (n = 2), hospital acquired infection (n = 1), measles (n = 2), viral hepatitis (n = 1), pyomyositis/cellulitis (n = 4).

^§^Acute otitis media (n = 2), bacterial conjunctivitis (n = 1), urinary tract infection (n = 2).

^**^Cases without identified source of infection for the fever on clinical and/or laboratory investigations conducted.

^**¶**^Comparison of proportions between all health centres and HUCSH.

*Significant difference (p-value < 0.05 from chi-square test).

### Clinical management

Table 4 summarizes the clinical management and outcomes of febrile illnesses in different health facilities. Of 338 children at health centres, 5 (1.5%) were referred to a higher-level facility for further care, while 138 (70.4%) of 196 at HUCSH were hospitalized at initial management on enrolment day. Of those hospitalized, 94 (68.1%) were due to pneumonia.

**Table 4:** Clinical management and outcomes of febrile children attending HUCSH and health centres in Hawassa City, 2019.

Characteristics	Health centres				HUCSH n (%)	p-value^¶^
	Tilte HC n (%)	Finchawa HC n (%)	Gara-Rikata HC n (%)	Total (HCs) n (%)		
**Initial management**	**N = 138**	**N = 100**	**N = 100**	**N = 338**	**N = 196**	
Managed as inpatient	–	–	–	–	138 (70.4)	
Managed as outpatient	135 (97.8)	100 (100)	98 (98.0)	333 (98.5)	58 (29.6)	
Referred to a higher-level care	3 (2.2)	0 (0)	2 (2.0)	5 (1.5)	0 (0)	
**Prescriptions and adherence with guidelines**						
Prescribed antibacterials	94 (68.1)	45 (45.0)	45 (45.0)	184 (54.4)	182 (92.9)	< 0.001*
Without indication for antibacterials	50 (36.2)	65 (65.0)	59(59.0)	174 (51.5)	21 (10.7)	–
Overprescribed antibacterials^1^	6 (12.0)^a^	10 (15.4)^b^	4 (6.8)^c^	20 (11.5)^d^	7 (33.3)^e^	0.013**
Prescribed antimalarials	33 (23.9)	55 (55.0)	7 (7.0)	95 (28.1)	24 (12.2)	< 0.001*
Without indication for antimalarials	105 (76.1)	45 (45.0)	93 (93.0)	243 (71.9)	185 (94.4)	–
Overprescribed antimalarials^2^	0 (0)	0 (0)	0 (0)	0 (0)	13 (7.0) ^f^	–
Prescribed antibacterials and antimalarials	5 (3.6)	5 (5.0)	0 (0)	10 (3.0)	15 (7.7)	0.013*

	N = 134	N = 96	N = 97	N = 327	N = 192	
**Day 7(± 1) follow-up**						
Child status						
Live	134 (100)	96 (100)	97 (100)	327 (100)	190 (99.0)	–
Died	0 (0)	0 (0)	0 (0)	0 (0)	2 (1.0)	
Fever status						
Resolved	134 (100)	95 (99.0)	97 (100)	326 (99.7)	177 (93.2)^g^	< 0.001*
Persisted	0 (0)	1 (1.0)	0 (0)	1 (0.3)	13 (6.8)^g^	
Further action after first visit						
No further action taken	131 (97.8)	89 (92.7)	95 (97.9)	315 (96.3)	180 (94.7)	–
Unscheduled re-consultation	0 (0)	6 (6.3)	0 (0)	6 (1.8)	0 (0)	
Visited other facilities	3 (2.2)	1 (1.0)	2 (2.1)	6 (1.8)	9 (4.7)	
Self-medication	0 (0)	0 (0)	0 (0)	0	1 (0.5)	

*HC* health centre, *HUCSH* Hawassa University Comprehensive Specialized Hospital.

^**¶**^Comparison of proportions between all health centres and HUCSH.

^1^Among children without indication for antibacterial treatment according to guidelines.

^2^Among children without indication for antimalarial treatment (without laboratory confirmation of malaria).

^a^(N = 50), ^b^(N = 65), ^c^(N = 59), ^d^(N = 174), ^e^(N = 21), ^f^(N = 185), ^f^(N = 190).

*Significant difference (p-value < 0.05 from chi-square test); **Significant difference (p-value < 0.05 from Fisher’s exact test).

Antibacterial agents were prescribed to 184 (54.4%) of 338 children at health centres compared to 182 (92.9%) of 196 children managed at HUCSH (p < 0.001). Among 174 children without indication for antibacterial therapy at health centres, 20 (11.5%) received the treatment, as did 7 (33.3%) of 21 at HUCSH (p = 0.013). Of those 20 children, 8 (40.0%) had unspecified upper respiratory infections, 9 (45.0%) had acute diarrhoea, and 3 (15.0%) had undifferentiated fever.

For antimalarials, 95 (28.1%) of 338 children at health centres were prescribed; all of which met guidelines. Whereas, at HUCSH, antimalarials were prescribed to 24 (12.2%) of 196 children, including 13 (7.0%) of 185 children without laboratory confirmation of malaria (Table 4).

### Clinical outcomes on follow-up

Clinical outcome data were unavailable for 11 (3.3%) of 338 children at health centres and 5 (2.5%) of 197 children at HUCSH. Of 327 children managed at health centres, 5 were hospitalized elsewhere: 2 children were referred to higher-level care, and 3 were initially sent home. Of 192 children at HUCSH contacted on day 7 (± 1), 136 (70.8%) had been hospitalized on initial management, with a median (IQR) stay of 5 (4–7) days, while one child initially managed as an outpatient had been admitted by follow-up.

The fever resolved by day 7 in 326 (99.7%) of 327 children managed at health centres and 177 (93.2%) of 190 seen at HUCSH (p < 0.001). The median (IQR) time to resolution of fever from the baseline visit was 1 (1–2) day in children at health centres and 2 (1.8–3) days in those at HUCSH. Two (1.5%) of 136 inpatients at HUCSH died: one with pneumonia and acute diarrhoea and the other with acute diarrhoea (Table 4). Within the follow-up period, 9 (4.7%) of 192 children at HUCSH and 6 (1.8%) of 327 at health centres visited another or the same facility.

## Discussion

Our analysis identified several differences between presenting febrile illness, clinical management, and outcomes in primary- and tertiary-level health facilities and rural and urban settings. Pneumonia was the most common clinical diagnosis at the tertiary hospital (HUCSH), while malaria was prevalent at lower-level health facilities in both urban (Tilte) and rural (Finchawa) settings located closer to Lake Hawassa, in contrast to a rural setting further from the lake (Gara-Rikata). We also observed that children presenting at the hospital were frequently overprescribed antibacterials and antimalarials.

Our study has the strength of being the first comprehensive analysis in Ethiopia, and one of the few such studies in Africa, to assess clinical management of childhood febrile illnesses based on guidelines and clinical outcomes. However, this study was not without limitations. First, although we enrolled children from several geographical and health system contexts, we did not sample randomly from across the city and outside clinical settings. So, there may have been selection bias associated with the specific facilities chosen. Second, our study focused on evaluating whether health workers adhered to the existing national guidelines in prescribing antimicrobials; not on whether the guidelines are appropriate. However, further laboratory assessments (for example, for bacteraemia) would have provided further insight into the appropriateness of the guidelines, but were unavailable within the resource constraints of our study. Third, the study was conducted during a major malaria transmission period; thus, patterns of presenting symptoms/diagnoses might be different in other seasons. Fourth, there might be a recall or other bias relating to caregivers’ reports on children’s symptoms at enrolment or follow-up. Last, healthcare worker practices may have been influenced by the study itself, noting that peers were trained to collect data.

The higher proportion of pneumonia at HUCSH and associated admissions (68.1%) indicate referrals from lower-level facilities and attracting severely ill children. The finding at health centres (30.8%) was comparable with the proportion of pneumonia (34.0%) reported in a systematic review and meta-analysis from East Africa^27^. Our results reflect the need for continuing improvements in pneumonia control, including enhancing nutritional and hygiene status, and increasing immunization coverage in the administrative region from the present low level of 38%^22^, particularly vaccines against pneumococcus and *Haemophilus influenzae* type b^28^. Further, improving early care seeking is essential to reduce hospitalization, as a delay in seeking care at primary care health facilities was observed to increase the odds of admission among children seen at the hospital^23^.

Our findings suggest substantial local variability in malaria transmission, with higher proportions in children attending lower-level health facilities closer to Lake Hawassa. Residential proximity to breeding sites for *Anopheles* mosquitoes might contribute to the observed differences. Other reports from Ethiopia have shown a significant heterogeneity in the risk of malaria infection between and even within villages^29,30^, with those living closer to mosquito breeding sites at increased risk^29,30^. The observed lower proportion of malaria at the hospital, also close to the lake, may reflect the hospital’s region-wide referral role, attracting cases of fever-related diseases other than malaria. It is also possible that caregivers were more likely to seek care from health centres when they suspected malaria, as observed in the current study in which shivering was a commonly reported symptom at health centres than that at the hospital. The effective treatment of malaria at lower-level health facilities also reduces cases appearing at the hospital. Our result of a lower proportion (3.2%) of malaria in a similar study at the same hospital from May 2018 through February 2019^18^ may reflect seasonality, as the earlier study was conducted over 10 months while the current study was conducted during a major malaria transmission season. Moreover, every study participant was screened for malaria in the earlier study, whereas testing in the hospital in the current study was at the discretion of attending clinicians. Whereas, screening was provided for every febrile child at the health centres, as recommended by the World Health Organization for malaria high-risk areas^10^. The observed high proportions of malaria in some localities emphasize the need for intensifying the implementation of malaria control interventions, including the utilization of insecticide-treated mosquito nets, indoor residual spraying, and early diagnosis and treatment, particularly at malaria hotspots.

The prescribing of antimalarial agents at health centres strictly followed national guidelines, in contrast to the proportion of overprescribing antimalarials (7.0%) at the hospital, as was reported in our earlier study from the same hospital (7.3%)^19^. Other studies from African countries showed that antimalarials were overprescribed to patients negative for malaria (11.5–58%)^12–14^ or without blood testing (16 and 42.7%)^13,14^, particularly to those seen at hospitals and inpatients^14^. Similar to our observation, greater adherence to malaria treatment guidelines by lower-level health workers was previously reported^12^, which may be due to clinicians with higher levels of training and experience being likely to make treatment decisions based on patient symptoms. There is also a report from Uganda on overprescribing antibacterials (42%)^15^ to patients without clinical indications, as shown in our current (33.3%) and earlier studies (34.0%)^19^. Most febrile children who had not received antibacterials or antimalarials recovered from their illnesses, reflecting the appropriateness of withholding medications when not indicated by guidelines. However, the guidelines, which recommend empiric antibacterial therapy for several clinically defined conditions, including pneumonia and tonsillopharyngitis, need to be improved by introducing diagnostic tools that can determine bacterial causes, as viral agents are also associated with these illnesses^31,32^.

Our results of resolution of fever (99.7%) by day 7 at health centres compared to that at the hospital (93.2%) were possibly associated with severity status as reflected by hospitalization and multiple symptom and signs. The observed proportion of resolved fever was consistent with our earlier result from the hospital (89.7%)^19^ and with studies elsewhere (89–98%)^33–35^. No death was reported among children enrolled at health centres, again consistent with reports from other countries^34,36,37^. Our observed in-hospital fatality ratio of 1.5% at HUCSH was similar to a result in Tanzania (1.4%)^38^ although contrasting higher fatality ratios were shown in the same country (5.7% and 7.3%)^39,40^ and in our earlier study (5.9%)^19^. In light of the observed outcomes, the clinical management of childhood febrile illnesses, both at lower- and higher-level health facilities, is mostly successful, encouraging caregivers to utilize care at lower-health facilities at the early stage of illness.

## Conclusion

The high proportion of pneumonia calls for intensified pneumonia control, particularly ensuring early care seeking and treatment at the community level to reduce associated hospitalization. Heterogeneous malaria transmission in the study sites highlights the need for strengthening interventions at high-risk localities to optimize resources and progress in malaria elimination. We suggest opportunities for optimizing the use of antimicrobials by integrating diagnostic tools to determine bacterial causes of febrile illness and enhancing health workers’ adherence to guidelines in lower- and higher-level health facilities.

## Supplementary Information

Supplementary Information 1.

Supplementary Table S1.

## Data Availability

The datasets generated and/or analysed during the current study are available from the corresponding author, upon reasonable request and with permission of the UNSW Human Research Ethics and the Institutional Review Board of the Hawassa University College of Medicine and Health Sciences. Restrictions apply to the availability of these potentially sensitive clinical data, which caregivers had consented for the collected information to be used for the purpose of our research study only, and so are not publicly available.
