# Supplementary material for: Differences in diagnosis, management, and outcomes of acute febrile illness by health facility level in southern Ethiopia
Source: Sci Rep. 2022 Nov 10;12:19166. doi: 10.1038/s41598-022-23641-8 (PMC9649757; doi:10.1038/s41598-022-23641-8)
Supplement: Supplementary file 2 — Supplementary Table S1. [file 41598_2022_23641_MOESM2_ESM.docx]

Table S1: Common diagnoses considered to be indications for antibacterial according to national management guidelines.

| Antibacterial treatment indicated | Antibacterial treatment not indicated^3^ |
| --- | --- |
| Tonsilopharyngitis | Upper respiratory infections (non-specified) |
| Pneumonia | Malaria |
| Lower respiratory infections (non-specified) | Intestinal parasites |
| Urinalysis findings suggestive of urinary tract infections (presence of pyuria/leucocyte esterase/nitrite)^1^ | Gastrointestinal bleeding- non infective |
| Dysentery or stool findings suggestive of enteric bacterial infections (presence of fecal pus cells and red blood cells)^1^ | Non-dysenteric diarrhoea without stool findings suggestive of enteric bacterial infections |
| Blood leukocytosis suggestive of bacterial infection^1^ | Non-severe febrile illness (non-specified) |
| Meningitis | Human immunodeficiency virus^4^ |
| Sepsis | Mumps |
| Endocarditis | Fungal infections |
| Acute otitis media | Viral hepatitis |
| Bacterial conjunctivitis | Measles |
| Pyogenic lymphadenitis | Anaemia |
| Abscess/infected tissue | Reactive airway disease/asthma |
| Appendiceal mass | Febrile seizure |
| Severe febrile disease (non-specified)^2^ | Flaccid paralysis |
| Acute glomerulonephritis | Scabies |
| Pyomyositis | Dermatitis |
| Severe acute malnutrition | Allergies |
| Peritonitis | Epilepsy |
| Intussusception | Cushing syndrome |
| Culture confirmed bacteraemia | Rickets |
| Culture confirmed urinary tract infection | Down syndrome |
| Culture confirmed shigellosis | Goitre |
| Culture confirmed salmonellosis | Mild/moderate malnutrition |

^1^ Clinicians’ discretion to withhold antibacterial agents among these cases was not considered as non-adherence if further examination was sought.

^2^ Severity of illness is based on the requirement for hospital admission.

^3^ However, antibacterial treatment is indicated if these conditions co-exist with those specified in the left-hand column.

^4^ Human immunodeficiency virus is not an indication for antibacterial treatment, but antibacterial prophylaxis may be prescribed, which is appropriate.
